# Bis(*N*-isopropyl-*N*-methyl­dithio­carbamato-κ^2^ *S*,*S*′)(1,10-phenanthroline-κ^2^ *N*,*N*′)zinc

**DOI:** 10.1107/S1600536811012499

**Published:** 2011-04-07

**Authors:** Nor Asiken Abdul Wahab, Ibrahim Baba, Mohamed Ibrahim Mohamed Tahir, Edward R. T. Tiekink

**Affiliations:** a School of Chemical Sciences and Food Technology, Faculty of Science and Technology, Universiti Kebangsaan Malaysia, 43600 Bangi, Malaysia; b Department of Chemistry, Universiti Putra Malaysia, 43400 Serdang, Malaysia; c Department of Chemistry, University of Malaya, 50603 Kuala Lumpur, Malaysia

## Abstract

The Zn^II^ atom in the title compound, [Zn(C_5_H_10_NS_2_)_2_(C_12_H_8_N_2_)], exists in a distorted *cis*-octa­hedral N_2_S_4_ donor set defined by two chelating dithio­carbamate anions as well as a 1,10-phenanthroline ligand. Each of the ligands coordinates in a symmetric mode. The crystal packing is stabilized by weak C—H⋯S, C—H⋯π(ZnS_2_C) and π–π [ring centroid distance between centrosymmetrically related pyridyl rings = 3.5955 (13) Å] inter­actions.

## Related literature

For the use of the parent zinc compound and nitro­gen adducts as precursors for ZnS nanoparticles, see: Motevalli *et al.* (1996 ▶); Malik *et al.* (1997 ▶). For background to supra­molecular polymers of zinc-triad dithio­carbamates and related structures, see: Benson *et al.* (2007 ▶); Jamaluddin *et al.* (2011 ▶). For a description of C—H⋯π(*M*S_2_C) inter­actions, see: Tiekink & Zukerman-Schpector (2011 ▶).
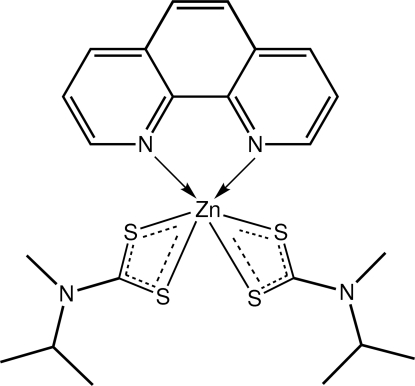


## Experimental

### 

#### Crystal data

[Zn(C_5_H_10_NS_2_)_2_(C_12_H_8_N_2_)]
*M* *_r_* = 542.09
Monoclinic, 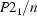

*a* = 11.8015 (3) Å
*b* = 16.6316 (4) Å
*c* = 13.7505 (3) Å
β = 101.738 (2)°
*V* = 2642.48 (11) Å^3^
*Z* = 4
Mo *K*α radiation
μ = 1.26 mm^−1^
*T* = 150 K
0.25 × 0.20 × 0.12 mm

#### Data collection

Oxford Diffraction Xcaliber Eos Gemini diffractometer
Absorption correction: multi-scan (*CrysAlis PRO*; Oxford Diffraction, 2010 ▶) *T*_min_ = 0.777, *T*_max_ = 0.860
33394 measured reflections
6001 independent reflections
4814 reflections with *I* > 2σ(*I*)
*R*_int_ = 0.064

#### Refinement

*R*[*F* ^2^ > 2σ(*F* ^2^)] = 0.036
*wR*(*F* ^2^) = 0.093
*S* = 1.04
6001 reflections
286 parameters
6 restraints
H-atom parameters constrained
Δρ_max_ = 0.66 e Å^−3^
Δρ_min_ = −0.50 e Å^−3^

### 

Data collection: *CrysAlis PRO* (Oxford Diffraction, 2010 ▶); cell refinement: *CrysAlis PRO*; data reduction: *CrysAlis PRO*; program(s) used to solve structure: *SHELXS97* (Sheldrick, 2008 ▶); program(s) used to refine structure: *SHELXL97* (Sheldrick, 2008 ▶); molecular graphics: *ORTEP-3* (Farrugia, 1997 ▶) and *DIAMOND* (Brandenburg, 2006 ▶); software used to prepare material for publication: *publCIF* (Westrip, 2010 ▶).

## Supplementary Material

Crystal structure: contains datablocks global, I. DOI: 10.1107/S1600536811012499/hb5835sup1.cif

Structure factors: contains datablocks I. DOI: 10.1107/S1600536811012499/hb5835Isup2.hkl

Additional supplementary materials:  crystallographic information; 3D view; checkCIF report

## Figures and Tables

**Table 1:** Selected bond lengths (Å)

Zn—S1	2.4782 (6)
Zn—S2	2.5408 (7)
Zn—S3	2.5031 (6)
Zn—S4	2.5132 (7)
Zn—N3	2.1939 (18)
Zn—N4	2.1970 (19)

**Table 2:** Hydrogen-bond geometry (Å, °) *Cg*1 is the centroid of the Zn,S1,S2,C1 chelate ring.

*D*—H⋯*A*	*D*—H	H⋯*A*	*D*⋯*A*	*D*—H⋯*A*
C7—H7b⋯S2^i^	0.98	2.79	3.734 (3)	162
C13—H13⋯S4^ii^	0.95	2.82	3.634 (2)	145
C21—H21⋯S1^iii^	0.95	2.84	3.684 (3)	149
C20—H20⋯*Cg*1^iv^	0.95	2.74	3.687 (2)	173

Symmetry codes: (i) 
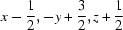
; (ii) 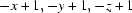; (iii) 
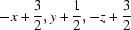
; (iv) 
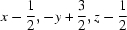
.

**Fig. 1.**
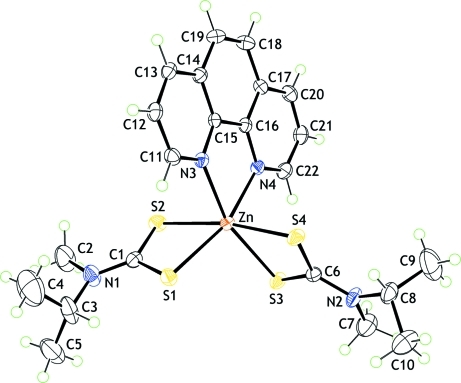
The molecular structure of of (I) showing displacement ellipsoids at the 50% probability level.

**Fig. 2.**
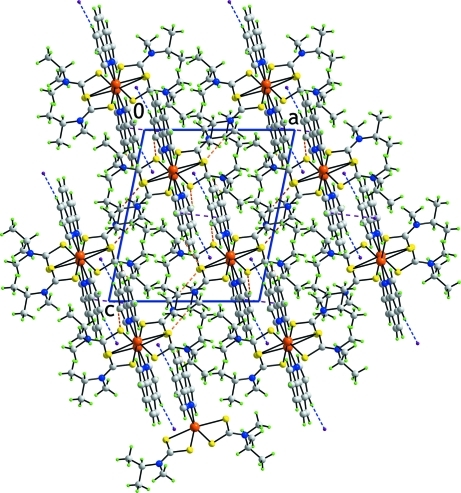
A view in projection down the b axis of the unit-cell contents for (I). The intermolecular C—H···S, C—H···π(ZnS2C) and π–π contacts are shown as orange, blue and purple dashed lines, respectively.

## supplementary crystallographic information

### Comment

The title compound Zn[S_2_CN(Me)iPr)_2_]_2_(1,10-phenanthroline), (I), was investigated as a part of on-going studies of zinc-triad dithiocarbamates and their adducts (Benson *et al.*, 2007; Jamaluddin *et al.*, 2011). The dinuclear parent {Zn[S_2_CN(Me)iPr]_2_}_2_ compound and its nitrogen-based adducts have proven useful as synthetic precursors for ZnS nanoparticles (Motevalli *et al.*, 1996; Malik *et al.*, 1997).

The Zn atom in (I), Fig. 1, is chelated by two symmetrically coordinating dithiocarbamate ligands, Table 1, and also symmetrically by the 1,10-phenanthroline ligand. The symmetric mode of coordination of the dithiocarbamate ligands is reflected in the narrow range of associated C≐ S bond distances, *i.e*. 1.718 (2) to 1.724 (2) Å, which are in fact experimentally equivalent. The N_2_S_4_ donor set defines a distorted octahedron with distortions readily explained in terms of the restricted bite distances of the chelating ligands.

The crystal structure is stabilized by weak intermolecular interactions. These include C—H···S and C—H···π(ZnS_2_C), Table 2, and π–π interactions. The C—H···π(ZnS_2_C) contacts have precedents in the crystal chemistry of metal dithiocarbamates (Tiekink & Zukerman-Schpector, 2011). The π–π interactions occur between centrosymmetrically related pyridyl rings [ring centroid(N3,C11–C15)···ring centroid(N3,C11–C15)^i^ = 3.5955 (13) Å for *i*: 1 - *x*, 1 - *y*, 1 - *z*]. A view of the unit-cell contents is shown in Fig. 2 where it can be seen that globally, the crystal packing comprises alternating layers of ZnS_2_CN/1,10-phenanthroline residues and alkyl groups.

### Experimental

The title compound was prepared using an *in situ* method by the addition of carbon disulfide (0.02 mol) to an ethanolic solution (20 ml) of isopropropyl(methyl)amine (0.02 mol) and 2,2'-bipyridine (0.01 mol) in ethanol (20 ml). The mixture was stirred for 1 h at 277 K. The resulting solution was added drop-wise to a solution of zinc(II) dichloride (0.01 mol) in ethanol (20 ml). The mixture was stirred for a further 2 h. The yellow precipitate was filtered and washed with cold ethanol, and dried in a desiccator. Crystallization was carried using an ethanol:chloroform (1:2 *v*/*v*) solvent system to yield pale yellow prisms of (I); *M*.pt. 420–421 K. Elemental analysis. Found (calculated) for C_22_H_32_CdN_4_S_4_: C, 46.49 (46.36); H 5.13 (5.45); N 10.74 (10.81) %. UV (CHCl_3_) λ_max_ 306.5 nm (*L*(π) →*L*(π*)). IR (KBr): ν(C—H) 2928 s; ν(C≐N) 1564 s; ν(N—C) 1473 s; ν(C≐S) 976 s; ν(Cd—S) 384 s cm^-1^.

### Refinement

Carbon-bound H-atoms were placed in calculated positions (C—H 0.95 to 1.00 Å) and were included in the refinement in the riding model approximation, with *U*_iso_(H) set to 1.2 to 1.5*U*_equiv_(C). Disorder was noted in the *N*-alkyl groups of both dithiocarbamate ligands. However, multiple sites could not be resolved. The C7 atom was refined with the ISOR command in *SHELX76* (Sheldrick, 2008) in order to obtain a reasonable displacement ellipsoid. The crystallographic assignment of atom types (in response to a level B alert concerning a Hirshfeld test difference for the N2—C7 bond) was substantiated by the elemental analysis and spectroscopy.

### Crystal data

**Table:**

[Zn(C_5_H_10_NS_2_)_2_(C_12_H_8_N_2_)]	*F*(000) = 1128
*M**_r_* = 542.09	*D*_x_ = 1.363 Mg m^−^^3^
Monoclinic, *P*2_1_/*n*	Mo *K*α radiation, λ = 0.71073 Å
Hall symbol: -P 2yn	Cell parameters from 11977 reflections
*a* = 11.8015 (3) Å	θ = 2–29°
*b* = 16.6316 (4) Å	µ = 1.26 mm^−^^1^
*c* = 13.7505 (3) Å	*T* = 150 K
β = 101.738 (2)°	Prism, pale-yellow
*V* = 2642.48 (11) Å^3^	0.25 × 0.20 × 0.12 mm
*Z* = 4	

### Data collection

**Table:**

Oxford Diffraction Xcaliber Eos Gemini diffractometer	6001 independent reflections
Radiation source: fine-focus sealed tube	4814 reflections with *I* > 2σ(*I*)
graphite	*R*_int_ = 0.064
Detector resolution: 16.1952 pixels mm^-1^	θ_max_ = 27.5°, θ_min_ = 2.2°
ω scans	*h* = −15→15
Absorption correction: multi-scan (*CrysAlis PRO*; Oxford Diffraction, 2010)	*k* = −21→21
*T*_min_ = 0.777, *T*_max_ = 0.860	*l* = −17→17
33394 measured reflections	

### Refinement

**Table:**

Refinement on *F*^2^	Primary atom site location: structure-invariant direct methods
Least-squares matrix: full	Secondary atom site location: difference Fourier map
*R*[*F*^2^ > 2σ(*F*^2^)] = 0.036	Hydrogen site location: inferred from neighbouring sites
*wR*(*F*^2^) = 0.093	H-atom parameters constrained
*S* = 1.04	*w* = 1/[σ^2^(*F*_o_^2^) + (0.0376*P*)^2^ + 1.5727*P*] where *P* = (*F*_o_^2^ + 2*F*_c_^2^)/3
6001 reflections	(Δ/σ)_max_ = 0.001
286 parameters	Δρ_max_ = 0.66 e Å^−^^3^
6 restraints	Δρ_min_ = −0.50 e Å^−^^3^

### Special details

**Table:**

Geometry. All s.u.'s (except the s.u. in the dihedral angle between two l.s. planes) are estimated using the full covariance matrix. The cell s.u.'s are taken into account individually in the estimation of s.u.'s in distances, angles and torsion angles; correlations between s.u.'s in cell parameters are only used when they are defined by crystal symmetry. An approximate (isotropic) treatment of cell s.u.'s is used for estimating s.u.'s involving l.s. planes.
Refinement. Refinement of *F*^2^ against ALL reflections. The weighted *R*-factor *wR* and goodness of fit *S* are based on *F*^2^, conventional *R*-factors *R* are based on *F*, with *F* set to zero for negative *F*^2^. The threshold expression of *F*^2^ > 2σ(*F*^2^) is used only for calculating *R*-factors(gt) *etc*. and is not relevant to the choice of reflections for refinement. *R*-factors based on *F*^2^ are statistically about twice as large as those based on *F*, and *R*- factors based on ALL data will be even larger.

### Fractional atomic coordinates and isotropic or equivalent isotropic displacement parameters (Å^2^)

**Table:**

	*x*	*y*	*z*	*U*_iso_*/*U*_eq_	
Zn	0.74542 (2)	0.617296 (16)	0.733396 (19)	0.02087 (9)	
S1	0.88167 (5)	0.51904 (4)	0.82966 (4)	0.02410 (14)	
S2	0.94125 (6)	0.63431 (4)	0.68609 (4)	0.02614 (15)	
S3	0.74451 (5)	0.71890 (4)	0.86753 (4)	0.02513 (15)	
S4	0.57158 (5)	0.59097 (4)	0.80817 (4)	0.02474 (14)	
N1	1.08885 (19)	0.52534 (15)	0.78012 (17)	0.0349 (5)	
N2	0.55956 (18)	0.70121 (13)	0.94727 (15)	0.0289 (5)	
N3	0.68974 (16)	0.53855 (11)	0.60453 (13)	0.0188 (4)	
N4	0.65988 (16)	0.69879 (11)	0.61528 (14)	0.0206 (4)	
C1	0.9824 (2)	0.55502 (15)	0.76623 (17)	0.0239 (5)	
C2	1.1701 (3)	0.5547 (2)	0.7204 (3)	0.0553 (9)	
H2A	1.1340	0.5512	0.6497	0.083*	
H2B	1.2404	0.5218	0.7338	0.083*	
H2C	1.1901	0.6108	0.7377	0.083*	
C3	1.1290 (3)	0.45930 (18)	0.8512 (2)	0.0450 (8)	
H3	1.0679	0.4513	0.8911	0.054*	
C4	1.1391 (4)	0.3815 (2)	0.7977 (4)	0.0847 (14)	
H4A	1.0650	0.3692	0.7534	0.127*	
H4B	1.1594	0.3380	0.8463	0.127*	
H4C	1.1995	0.3865	0.7586	0.127*	
C5	1.2390 (3)	0.4817 (2)	0.9235 (3)	0.0609 (10)	
H5A	1.3038	0.4822	0.8888	0.091*	
H5B	1.2543	0.4422	0.9774	0.091*	
H5C	1.2302	0.5351	0.9510	0.091*	
C6	0.6178 (2)	0.67345 (14)	0.88098 (16)	0.0214 (5)	
C7	0.5997 (3)	0.77392 (18)	1.0047 (2)	0.0433 (7)	
H7A	0.6762	0.7640	1.0463	0.065*	
H7B	0.5450	0.7878	1.0470	0.065*	
H7C	0.6046	0.8185	0.9591	0.065*	
C8	0.4491 (2)	0.66584 (18)	0.9609 (2)	0.0331 (6)	
H8	0.4375	0.6144	0.9225	0.040*	
C9	0.3502 (3)	0.7211 (2)	0.9181 (3)	0.0684 (11)	
H9A	0.3586	0.7718	0.9551	0.103*	
H9B	0.2768	0.6956	0.9234	0.103*	
H9C	0.3507	0.7317	0.8481	0.103*	
C10	0.4521 (3)	0.6458 (2)	1.0693 (2)	0.0504 (8)	
H10A	0.5227	0.6155	1.0964	0.076*	
H10B	0.3843	0.6133	1.0743	0.076*	
H10C	0.4513	0.6957	1.1071	0.076*	
C11	0.7038 (2)	0.45954 (14)	0.60029 (17)	0.0229 (5)	
H11	0.7385	0.4319	0.6593	0.027*	
C12	0.6698 (2)	0.41511 (14)	0.51280 (17)	0.0231 (5)	
H12	0.6806	0.3585	0.5130	0.028*	
C13	0.6210 (2)	0.45394 (14)	0.42725 (17)	0.0216 (5)	
H13	0.5981	0.4246	0.3672	0.026*	
C14	0.60452 (19)	0.53794 (14)	0.42823 (16)	0.0196 (5)	
C15	0.64063 (18)	0.57743 (13)	0.51983 (16)	0.0168 (4)	
C16	0.62276 (19)	0.66277 (13)	0.52604 (16)	0.0176 (5)	
C17	0.56638 (19)	0.70521 (14)	0.44123 (16)	0.0200 (5)	
C18	0.5324 (2)	0.66342 (15)	0.34901 (17)	0.0254 (5)	
H18	0.4959	0.6921	0.2914	0.031*	
C19	0.5516 (2)	0.58322 (15)	0.34272 (16)	0.0245 (5)	
H19	0.5295	0.5568	0.2805	0.029*	
C20	0.5474 (2)	0.78788 (14)	0.45176 (18)	0.0243 (5)	
H20	0.5083	0.8185	0.3968	0.029*	
C21	0.5854 (2)	0.82358 (15)	0.54158 (19)	0.0270 (5)	
H21	0.5734	0.8795	0.5497	0.032*	
C22	0.6422 (2)	0.77763 (14)	0.62167 (18)	0.0258 (5)	
H22	0.6694	0.8036	0.6835	0.031*	

### Atomic displacement parameters (Å^2^)

**Table:**

	*U*^11^	*U*^22^	*U*^33^	*U*^12^	*U*^13^	*U*^23^
Zn	0.02263 (16)	0.02294 (16)	0.01688 (14)	0.00262 (11)	0.00360 (10)	−0.00228 (11)
S1	0.0225 (3)	0.0270 (3)	0.0237 (3)	0.0021 (2)	0.0069 (2)	0.0075 (2)
S2	0.0278 (3)	0.0285 (3)	0.0229 (3)	−0.0016 (3)	0.0070 (2)	0.0066 (3)
S3	0.0233 (3)	0.0283 (3)	0.0248 (3)	−0.0056 (2)	0.0073 (2)	−0.0095 (3)
S4	0.0247 (3)	0.0272 (3)	0.0226 (3)	−0.0051 (2)	0.0054 (2)	−0.0087 (2)
N1	0.0247 (12)	0.0447 (14)	0.0387 (12)	0.0059 (10)	0.0144 (10)	0.0145 (11)
N2	0.0262 (12)	0.0332 (12)	0.0304 (11)	−0.0037 (9)	0.0131 (9)	−0.0135 (10)
N3	0.0195 (10)	0.0179 (10)	0.0192 (9)	0.0016 (8)	0.0045 (7)	−0.0008 (8)
N4	0.0206 (10)	0.0198 (10)	0.0213 (9)	0.0018 (8)	0.0039 (8)	−0.0047 (8)
C1	0.0251 (13)	0.0258 (13)	0.0217 (11)	−0.0010 (10)	0.0071 (9)	−0.0021 (10)
C2	0.0346 (18)	0.078 (3)	0.061 (2)	0.0119 (16)	0.0263 (15)	0.0265 (19)
C3	0.0325 (16)	0.0416 (18)	0.065 (2)	0.0149 (13)	0.0184 (14)	0.0241 (16)
C4	0.080 (3)	0.049 (2)	0.119 (4)	0.018 (2)	0.007 (3)	0.003 (2)
C5	0.041 (2)	0.078 (3)	0.061 (2)	0.0160 (18)	0.0045 (16)	0.027 (2)
C6	0.0208 (12)	0.0241 (13)	0.0191 (11)	−0.0006 (9)	0.0034 (9)	−0.0035 (9)
C7	0.0447 (11)	0.0438 (11)	0.0440 (10)	−0.0024 (8)	0.0150 (8)	−0.0098 (8)
C8	0.0276 (15)	0.0411 (16)	0.0346 (14)	−0.0039 (12)	0.0155 (11)	−0.0053 (12)
C9	0.036 (2)	0.089 (3)	0.084 (3)	0.0108 (19)	0.0224 (18)	0.031 (2)
C10	0.061 (2)	0.056 (2)	0.0398 (17)	−0.0082 (17)	0.0231 (15)	−0.0037 (15)
C11	0.0230 (13)	0.0221 (12)	0.0233 (11)	0.0035 (10)	0.0042 (9)	0.0040 (10)
C12	0.0241 (13)	0.0176 (12)	0.0282 (12)	0.0024 (9)	0.0070 (10)	−0.0021 (10)
C13	0.0216 (12)	0.0206 (12)	0.0236 (11)	−0.0012 (9)	0.0071 (9)	−0.0044 (10)
C14	0.0176 (12)	0.0210 (12)	0.0209 (11)	−0.0019 (9)	0.0053 (9)	−0.0024 (9)
C15	0.0141 (11)	0.0177 (11)	0.0191 (10)	0.0002 (9)	0.0048 (8)	0.0000 (9)
C16	0.0161 (11)	0.0167 (11)	0.0205 (11)	−0.0011 (9)	0.0049 (8)	−0.0014 (9)
C17	0.0170 (12)	0.0200 (12)	0.0222 (11)	0.0004 (9)	0.0022 (9)	0.0007 (9)
C18	0.0283 (14)	0.0245 (13)	0.0207 (11)	−0.0016 (10)	−0.0019 (10)	0.0023 (10)
C19	0.0281 (14)	0.0278 (14)	0.0164 (11)	−0.0034 (10)	0.0013 (9)	−0.0021 (10)
C20	0.0224 (13)	0.0212 (13)	0.0285 (12)	0.0014 (10)	0.0029 (10)	0.0043 (10)
C21	0.0286 (14)	0.0175 (12)	0.0364 (14)	0.0033 (10)	0.0104 (11)	−0.0019 (10)
C22	0.0296 (14)	0.0216 (13)	0.0262 (12)	0.0021 (10)	0.0058 (10)	−0.0060 (10)

### Geometric parameters (Å, °)

**Table:**

Zn—S1	2.4782 (6)	C7—H7A	0.9800
Zn—S2	2.5408 (7)	C7—H7B	0.9800
Zn—S3	2.5031 (6)	C7—H7C	0.9800
Zn—S4	2.5132 (7)	C8—C9	1.508 (4)
Zn—N3	2.1939 (18)	C8—C10	1.521 (4)
Zn—N4	2.1970 (19)	C8—H8	1.0000
S1—C1	1.719 (2)	C9—H9A	0.9800
S2—C1	1.724 (2)	C9—H9B	0.9800
S3—C6	1.718 (2)	C9—H9C	0.9800
S4—C6	1.720 (2)	C10—H10A	0.9800
N1—C1	1.326 (3)	C10—H10B	0.9800
N1—C2	1.467 (3)	C10—H10C	0.9800
N1—C3	1.483 (3)	C11—C12	1.399 (3)
N2—C6	1.331 (3)	C11—H11	0.9500
N2—C7	1.469 (3)	C12—C13	1.363 (3)
N2—C8	1.476 (3)	C12—H12	0.9500
N3—C11	1.327 (3)	C13—C14	1.411 (3)
N3—C15	1.355 (3)	C13—H13	0.9500
N4—C22	1.334 (3)	C14—C15	1.407 (3)
N4—C16	1.356 (3)	C14—C19	1.429 (3)
C2—H2A	0.9800	C15—C16	1.440 (3)
C2—H2B	0.9800	C16—C17	1.409 (3)
C2—H2C	0.9800	C17—C20	1.405 (3)
C3—C4	1.506 (5)	C17—C18	1.430 (3)
C3—C5	1.512 (5)	C18—C19	1.359 (4)
C3—H3	1.0000	C18—H18	0.9500
C4—H4A	0.9800	C19—H19	0.9500
C4—H4B	0.9800	C20—C21	1.362 (3)
C4—H4C	0.9800	C20—H20	0.9500
C5—H5A	0.9800	C21—C22	1.395 (3)
C5—H5B	0.9800	C21—H21	0.9500
C5—H5C	0.9800	C22—H22	0.9500
			
N3—Zn—N4	75.76 (7)	N2—C7—H7A	109.5
N3—Zn—S1	95.33 (5)	N2—C7—H7B	109.5
N4—Zn—S1	162.22 (5)	H7A—C7—H7B	109.5
N3—Zn—S3	162.30 (5)	N2—C7—H7C	109.5
N4—Zn—S3	93.33 (5)	H7A—C7—H7C	109.5
S1—Zn—S3	98.69 (2)	H7B—C7—H7C	109.5
N3—Zn—S4	95.23 (5)	N2—C8—C9	110.0 (2)
N4—Zn—S4	96.85 (5)	N2—C8—C10	111.5 (2)
S1—Zn—S4	99.30 (2)	C9—C8—C10	112.2 (3)
S3—Zn—S4	71.94 (2)	N2—C8—H8	107.7
N3—Zn—S2	89.90 (5)	C9—C8—H8	107.7
N4—Zn—S2	92.70 (5)	C10—C8—H8	107.7
S1—Zn—S2	71.65 (2)	C8—C9—H9A	109.5
S3—Zn—S2	104.71 (2)	C8—C9—H9B	109.5
S4—Zn—S2	170.02 (2)	H9A—C9—H9B	109.5
C1—S1—Zn	86.57 (8)	C8—C9—H9C	109.5
C1—S2—Zn	84.50 (8)	H9A—C9—H9C	109.5
C6—S3—Zn	85.22 (8)	H9B—C9—H9C	109.5
C6—S4—Zn	84.85 (8)	C8—C10—H10A	109.5
C1—N1—C2	120.2 (2)	C8—C10—H10B	109.5
C1—N1—C3	122.5 (2)	H10A—C10—H10B	109.5
C2—N1—C3	117.3 (2)	C8—C10—H10C	109.5
C6—N2—C7	119.9 (2)	H10A—C10—H10C	109.5
C6—N2—C8	122.8 (2)	H10B—C10—H10C	109.5
C7—N2—C8	117.2 (2)	N3—C11—C12	123.0 (2)
C11—N3—C15	118.04 (19)	N3—C11—H11	118.5
C11—N3—Zn	127.37 (15)	C12—C11—H11	118.5
C15—N3—Zn	114.51 (14)	C13—C12—C11	119.3 (2)
C22—N4—C16	117.9 (2)	C13—C12—H12	120.4
C22—N4—Zn	127.77 (15)	C11—C12—H12	120.4
C16—N4—Zn	114.32 (15)	C12—C13—C14	119.7 (2)
N1—C1—S1	121.99 (19)	C12—C13—H13	120.1
N1—C1—S2	120.82 (19)	C14—C13—H13	120.1
S1—C1—S2	117.15 (14)	C15—C14—C13	117.0 (2)
N1—C2—H2A	109.5	C15—C14—C19	119.5 (2)
N1—C2—H2B	109.5	C13—C14—C19	123.5 (2)
H2A—C2—H2B	109.5	N3—C15—C14	123.0 (2)
N1—C2—H2C	109.5	N3—C15—C16	117.49 (19)
H2A—C2—H2C	109.5	C14—C15—C16	119.45 (19)
H2B—C2—H2C	109.5	N4—C16—C17	122.6 (2)
N1—C3—C4	111.2 (3)	N4—C16—C15	117.71 (19)
N1—C3—C5	111.6 (3)	C17—C16—C15	119.66 (19)
C4—C3—C5	112.9 (3)	C20—C17—C16	117.6 (2)
N1—C3—H3	106.9	C20—C17—C18	123.0 (2)
C4—C3—H3	106.9	C16—C17—C18	119.4 (2)
C5—C3—H3	106.9	C19—C18—C17	120.9 (2)
C3—C4—H4A	109.5	C19—C18—H18	119.6
C3—C4—H4B	109.5	C17—C18—H18	119.6
H4A—C4—H4B	109.5	C18—C19—C14	121.0 (2)
C3—C4—H4C	109.5	C18—C19—H19	119.5
H4A—C4—H4C	109.5	C14—C19—H19	119.5
H4B—C4—H4C	109.5	C21—C20—C17	119.3 (2)
C3—C5—H5A	109.5	C21—C20—H20	120.3
C3—C5—H5B	109.5	C17—C20—H20	120.3
H5A—C5—H5B	109.5	C20—C21—C22	119.6 (2)
C3—C5—H5C	109.5	C20—C21—H21	120.2
H5A—C5—H5C	109.5	C22—C21—H21	120.2
H5B—C5—H5C	109.5	N4—C22—C21	122.9 (2)
N2—C6—S4	121.87 (19)	N4—C22—H22	118.5
N2—C6—S3	120.21 (18)	C21—C22—H22	118.5
S4—C6—S3	117.92 (14)		
			
N3—Zn—S1—C1	85.85 (10)	C2—N1—C3—C5	56.7 (4)
N4—Zn—S1—C1	27.03 (19)	C7—N2—C6—S4	−177.3 (2)
S3—Zn—S1—C1	−104.98 (8)	C8—N2—C6—S4	−2.4 (3)
S4—Zn—S1—C1	−177.95 (8)	C7—N2—C6—S3	3.8 (3)
S2—Zn—S1—C1	−2.27 (8)	C8—N2—C6—S3	178.72 (19)
N3—Zn—S2—C1	−93.38 (9)	Zn—S4—C6—N2	178.5 (2)
N4—Zn—S2—C1	−169.12 (9)	Zn—S4—C6—S3	−2.66 (13)
S1—Zn—S2—C1	2.27 (8)	Zn—S3—C6—N2	−178.4 (2)
S3—Zn—S2—C1	96.72 (8)	Zn—S3—C6—S4	2.67 (13)
S4—Zn—S2—C1	27.68 (16)	C6—N2—C8—C9	−106.7 (3)
N3—Zn—S3—C6	43.33 (19)	C7—N2—C8—C9	68.4 (3)
N4—Zn—S3—C6	94.43 (9)	C6—N2—C8—C10	128.3 (3)
S1—Zn—S3—C6	−98.71 (8)	C7—N2—C8—C10	−56.7 (3)
S4—Zn—S3—C6	−1.70 (8)	C15—N3—C11—C12	−0.3 (3)
S2—Zn—S3—C6	−171.90 (8)	Zn—N3—C11—C12	−176.88 (17)
N3—Zn—S4—C6	−165.83 (9)	N3—C11—C12—C13	0.7 (4)
N4—Zn—S4—C6	−89.58 (9)	C11—C12—C13—C14	−0.6 (3)
S1—Zn—S4—C6	97.88 (8)	C12—C13—C14—C15	0.1 (3)
S3—Zn—S4—C6	1.70 (8)	C12—C13—C14—C19	−178.4 (2)
S2—Zn—S4—C6	73.51 (16)	C11—N3—C15—C14	−0.2 (3)
N4—Zn—N3—C11	−179.2 (2)	Zn—N3—C15—C14	176.82 (17)
S1—Zn—N3—C11	16.4 (2)	C11—N3—C15—C16	178.2 (2)
S3—Zn—N3—C11	−125.9 (2)	Zn—N3—C15—C16	−4.8 (2)
S4—Zn—N3—C11	−83.45 (19)	C13—C14—C15—N3	0.3 (3)
S2—Zn—N3—C11	87.97 (19)	C19—C14—C15—N3	178.9 (2)
N4—Zn—N3—C15	4.09 (15)	C13—C14—C15—C16	−178.1 (2)
S1—Zn—N3—C15	−160.28 (15)	C19—C14—C15—C16	0.5 (3)
S3—Zn—N3—C15	57.4 (3)	C22—N4—C16—C17	−0.3 (3)
S4—Zn—N3—C15	99.85 (15)	Zn—N4—C16—C17	−179.72 (17)
S2—Zn—N3—C15	−88.72 (15)	C22—N4—C16—C15	−179.2 (2)
N3—Zn—N4—C22	177.8 (2)	Zn—N4—C16—C15	1.5 (3)
S1—Zn—N4—C22	−120.7 (2)	N3—C15—C16—N4	2.2 (3)
S3—Zn—N4—C22	11.9 (2)	C14—C15—C16—N4	−179.3 (2)
S4—Zn—N4—C22	84.1 (2)	N3—C15—C16—C17	−176.6 (2)
S2—Zn—N4—C22	−93.0 (2)	C14—C15—C16—C17	1.9 (3)
N3—Zn—N4—C16	−2.91 (15)	N4—C16—C17—C20	−0.9 (3)
S1—Zn—N4—C16	58.6 (3)	C15—C16—C17—C20	177.9 (2)
S3—Zn—N4—C16	−168.78 (15)	N4—C16—C17—C18	178.4 (2)
S4—Zn—N4—C16	−96.59 (15)	C15—C16—C17—C18	−2.8 (3)
S2—Zn—N4—C16	86.31 (15)	C20—C17—C18—C19	−179.3 (2)
C2—N1—C1—S1	176.6 (2)	C16—C17—C18—C19	1.4 (4)
C3—N1—C1—S1	−0.1 (4)	C17—C18—C19—C14	1.0 (4)
C2—N1—C1—S2	−5.7 (4)	C15—C14—C19—C18	−2.0 (4)
C3—N1—C1—S2	177.6 (2)	C13—C14—C19—C18	176.5 (2)
Zn—S1—C1—N1	−178.6 (2)	C16—C17—C20—C21	1.2 (3)
Zn—S1—C1—S2	3.58 (13)	C18—C17—C20—C21	−178.1 (2)
Zn—S2—C1—N1	178.6 (2)	C17—C20—C21—C22	−0.2 (4)
Zn—S2—C1—S1	−3.50 (12)	C16—N4—C22—C21	1.4 (4)
C1—N1—C3—C4	106.4 (3)	Zn—N4—C22—C21	−179.31 (18)
C2—N1—C3—C4	−70.4 (4)	C20—C21—C22—N4	−1.1 (4)
C1—N1—C3—C5	−126.5 (3)		

### Hydrogen-bond geometry (Å, °)

**Table:**

Cg1 is the centroid of the Zn,S1,S2,C1 chelate ring.

**Table:**

*D*—H···*A*	*D*—H	H···*A*	*D*···*A*	*D*—H···*A*
C7—H7b···S2^i^	0.98	2.79	3.734 (3)	162
C13—H13···S4^ii^	0.95	2.82	3.634 (2)	145
C21—H21···S1^iii^	0.95	2.84	3.684 (3)	149
C20—H20···Cg1^iv^	0.95	2.74	3.687 (2)	173

Symmetry codes: (i) *x*−1/2, −*y*+3/2, *z*+1/2; (ii) −*x*+1, −*y*+1, −*z*+1; (iii) −*x*+3/2, *y*+1/2, −*z*+3/2; (iv) *x*−1/2, −*y*+3/2, *z*−1/2.
